# Siren Songs of an Effortless Academy: The Misuse of Artificial Intelligence

**DOI:** 10.17533/udea.iee.v43n3e01

**Published:** 2025-10-17

**Authors:** Carmen de la Cuesta Benjumea

**Affiliations:** 1 RN, MSc, PhD. Honorary Collaborating Professor, Department of Health Psychology. Universidad de Alicante. Alicante, San Vicente del Raspeig, Spain. Instituto de Investigación Sanitaria y Biomédica de Alicante (ISABIAL), Alicante, Spain. Email: ccuesta@ua.es. Corresponding author. https://orcid.org/0000-0003-2160-392X Universidad de Alicante Department of Health Psychology Universidad de Alicante Instituto de Investigación Sanitaria y Biomédica de Alicante (ISABIAL) Alicante Spain ccuesta@ua.es

The Odyssey tells that Ulysses, on his journey to Ithaca, upon sighting the island of the Sirens, requested to be tied to the ship’s mast to avoid succumbing to the songs of the Sirens, songs that, if he heard them, would bring him a tragic destiny. The use some propose, and others make of generative artificial intelligence (generative AI) are Siren songs for researchers and anyone doing work at a given standard.

Blumer,^1^ in his treatise on symbolic interactionism, indicates that it is the use we make of something that makes the difference and not the manufacturers’ specifications or commercial promises. Generative AI is a new resource that some propose and promote for an effortless academy, and - herein - I want to stop without having to tie myself to the mast. I have gotten commercial messages offering me AI to analyze qualitative data, which talk about automatic coding, avatars to conduct interviews that can also speak different languages, quick and easy systematic reviews, and effortless document creation. In one of these messages, I am invited to imagine what it would be like to have interviewer avatars that adapt and learn in real time or automatically generate topics into long narratives. I did not dare imagine such.

Generative AI simulates knowing without understanding, displaying a façade of knowledge that fades when applied in practical or complex contexts. This is called Potemkin understanding.^2^ The term stems from the stratagem used by Russian general Potemkin to amaze Empress Catherine the Great and foreign ambassadors during a visit to Crimea in the late 18^th^ century. Fake structures or village decorations were set up. It is said that he even mobilized healthy -looking peasants and rented cattle to give an impression of progress and well-being in those rural areas. Thus, AI creates the illusion of comprehension. Be careful, the precision AI offers is not equivalent to understanding.

A journalist recently echoed the misuse of AI to draft texts.^3^ Motivated by questions made by people close to her about scientific findings in the health field, she tracked the coverage of the most famous topics of recent months. She ended up reading “tons” of news elaborated by AI systems. These were characterized by a stilted style, excessive adjectives, and using bombastic terms, and hackneyed filler words. She pointed out that articles “written” by AI, in addition to endangering journalistic work, could be inaccurate and contain conceptual errors. That is, they were untrustworthy. 

Artificial intelligence systems neither write, nor conceptualize, nor reason, or do not do so like humans. Thus, their misuse erodes the foundations of research, besides discrediting the author and breaking the trust of the medium where they are published. Due to foregoing, researchers, young and senior, must use Generative AI responsibly in research. A systematic review of 24 published articles has indicated that AI facilitates, supports, improves, and aid in processes such as the generation of ideas and the design of a study, the bibliography review, data management and their analysis, and the elaboration of the report. Warning: it does not say that IAs do these. The authors conclude that AI has potential and that the challenge lies in maintaining academic integrity and balancing its use with human interpretation.^4^ Hence, we are not to tie ourselves to a mast to avoid using it; rather, we must educate ourselves to do so properly. In qualitative research, this training is especially relevant because it is a craft, creative work, in which one must be more faithful to the spirit than to the letter. 

Artificial intelligence systems are predictive models based on existing data, so when used thoughtlessly in literature reviews, these end up parroting what is known about a topic.^5^ It is well known that ChatGPT and other similar generative AI models can help improve the writing of a document once it has been written and not before. Asking an AI to create a report from scratch is effortless academy, a journey to nowhere. A tragic fate from which one must stay away. 

Increasingly, qualitative analysis software includes Generative AI among its functions. And tests are under way to determine its contribution. For example, a comparative study between analysis produced by traditional means and that produced with AI indicates that a complementary relationship is necessary between AI use and researchers.^6^ Among the biggest challenges, the authors found how to manage the inconsistencies and hallucinations produced by AI, which is why they pointed to the need to carry out verification processes to maintain the validity of qualitative analysis. 

Regarding systematic reviews, I consider that today they help us find references, eliminate duplicates and perform mechanical tasks such as data entry and information management, but they do not evaluate or synthesize the findings of primary studies. A work about the use of avatars in human-AI interaction states that these have great potential to promote it.^7^ Nevertheless, given that AI users are free to create them according to their preferences, they can generate discrimination and social prejudices, reinforcing stereotypes and inequalities. This study recognizes that the design of AI avatars is an area that has not been thoroughly studied, and it is noted that it remains to be seen whether user perception of human-like AI avatars is equivalent to that of real humans. Hence, to generate qualitative data, for now and leaving aside essential issues like theoretical sampling, its use does not seem advisable. 

To end, AI is here to stay; its intelligent and ethical use depends on us all. It helps us to be more effective and efficient, but not to be researchers; we can only achieve this through a lot of study, effort, and perseverance. The rest are Siren songs.
